# Transcription Factors Bind Negatively Selected Sites within Human mtDNA Genes

**DOI:** 10.1093/gbe/evu210

**Published:** 2014-09-22

**Authors:** Amit Blumberg, Badi Sri Sailaja, Anshul Kundaje, Liron Levin, Sara Dadon, Shimrit Shmorak, Eitan Shaulian, Eran Meshorer, Dan Mishmar

**Affiliations:** ^1^Department of Life Sciences, Ben-Gurion University of the Negev, Beer Sheva, Israel; ^2^Department of Genetics, The Institute of Life Sciences, and The Edmond Lily Center for Brain Sciences (ELSC), The Hebrew University of Jerusalem, Edmond J. Safra Campus, Givat Ram, Israel; ^3^Department of Genetics, Stanford University; ^4^Department of Computer Science, Stanford University; ^5^Department of Biochemistry and Molecular Biology, IMRIC, The Hebrew University Medical School, Ein Karem, Jerusalem, Israel

**Keywords:** CEBPb, c-Jun, ChIP-seq, Jun-D, mitochondrial DNA, negative selection, transcription

## Abstract

Transcription of mitochondrial DNA (mtDNA)-encoded genes is thought to be regulated by a handful of dedicated transcription factors (TFs), suggesting that mtDNA genes are separately regulated from the nucleus. However, several TFs, with known nuclear activities, were found to bind mtDNA and regulate mitochondrial transcription. Additionally, mtDNA transcriptional regulatory elements, which were proved important in vitro, were harbored by a deletion that normally segregated among healthy individuals. Hence, mtDNA transcriptional regulation is more complex than once thought. Here, by analyzing ENCODE chromatin immunoprecipitation sequencing (ChIP-seq) data, we identified strong binding sites of three bona fide nuclear TFs (c-Jun, Jun-D, and CEBPb) within human mtDNA protein-coding genes. We validated the binding of two TFs by ChIP-quantitative polymerase chain reaction (c-Jun and Jun-D) and showed their mitochondrial localization by electron microscopy and subcellular fractionation. As a step toward investigating the functionality of these TF-binding sites (TFBS), we assessed signatures of selection. By analyzing 9,868 human mtDNA sequences encompassing all major global populations, we recorded genetic variants in tips and nodes of mtDNA phylogeny within the TFBS. We next calculated the effects of variants on binding motif prediction scores. Finally, the mtDNA variation pattern in predicted TFBS, occurring within ChIP-seq negative-binding sites, was compared with ChIP-seq positive-TFBS (CPR). Motifs within CPRs of c-Jun, Jun-D, and CEBPb harbored either only tip variants or their nodal variants retained high motif prediction scores. This reflects negative selection within mtDNA CPRs, thus supporting their functionality. Hence, human mtDNA-coding sequences may have dual roles, namely coding for genes yet possibly also possessing regulatory potential.

## Introduction

The control of mitochondrial DNA (mtDNA) transcription is considered to be solely governed by nuclear DNA (nDNA)-encoded factors. This machinery was long thought to involve the import of mitochondrial-dedicated nDNA-encoded factors, followed by their binding to the mtDNA promoters region, found within the main mtDNA noncoding region (D-Loop), although exceptions have been reported (reviewed in Bestwick and Shadel 2013). It was, therefore, thought that mitochondrial transcription is regulated independently of the nuclear genome.

Several groups isolated the minimal set of factors essential for in vitro mitochondrial transcription, namely mitochondrial transcription factor (TF) A, mitochondrial TF B2, and mitochondrial RNA polymerase (Falkenberg et al. 2002; Cotney et al. 2007; Shutt and Shadel 2010; Shi et al. 2012). Later efforts added the mitochondrial transcription termination factor (MTERF) family to this set (reviewed in Guja and Garcia-Diaz 2012). Moreover, in vitro transcription experiments showed that deletions and point mutations within the D-Loop dramatically reduced mtDNA transcription, thus supporting the crucial role of the D-Loop in mtDNA transcriptional regulation (Pham et al. 2006). Nevertheless, proteins of the MTERF family regulate mtDNA transcription through recognition sites found both within and outside the D-Loop (Yakubovskaya et al. 2010). As such, mtDNA transcriptional regulation is likely governed by a combination of the promoters with additional, nonpromoter sites.

Is it possible that mtDNA transcriptional regulation is more complex than once thought? Possibly, such regulation is not separated from the regulatory system of the nucleus, but rather exhibits some form of coregulation. Several pieces of evidence suggest that this may indeed be the case. First, mtDNA deletions overlapping mutations that severely affect in vitro transcription normally segregate among unaffected individuals lacking any phenotypic outcome (Bi et al. 2010). Second, a number of nDNA-encoded factors that regulate the transcription of nDNA-encoded subunits of the oxidative phosphorylation (OXPHOS) machinery, such as the cAMP Response Element-Binding protein, that is, CREB (De Rasmo et al. 2009), Estrogen Receptor (ER) beta but not ER alpha (Chen et al. 2004; Grober et al. 2011), and P43 (Casas et al. 1999), were identified within mammalian mitochondria and shown to directly regulate mtDNA transcription (reviewed in Leigh-Brown et al. 2010). Finally, the TF myocyte enhancer factor 2D (MEF2D) was recently shown to regulate the transcription of specific genes within the light mtDNA strand through a binding site distant from the D-loop (She et al. 2011). Taken together, these findings suggest that mtDNA transcriptional regulation involves multiple factors, some of which are bona fide nDNA-encoded TFs that are transported into mitochondria, where they bind to different regions throughout the mtDNA to directly affect mtDNA transcription. This supports the hypothesis that mtDNA transcription could be coregulated along with the main nDNA transcriptional system (Bar-Yaacov et al. 2012).

One could argue that unlike the nDNA, the mtDNA is a bacterial genomic relic that is regulated by a mixture of bacteria- and virus-like TFs and hence should be considered differently than the nDNA. Even if this is the case, distant regulatory elements, such as enhancers, have been identified in a variety of bacterial taxa (Xu and Hoover 2001). Hence, it can be hypothesized that the mtDNA harbors distant nonpromoter transcription regulatory elements. Therefore, a hypothesis-free approach is preferable for identification of novel mtDNA TF-binding sites (TFBS) in vivo.

Here, we performed a bioinformatics screen of chromatin immunoprecipitation sequencing (ChIP-seq) data from the ENCODE consortium (Myers et al. 2011; Encode-Project-Consortium et al. 2012), which enabled the identification and, in turn, experimental validation of the binding of bona fide nDNA TFs to novel sites distant from human mtDNA promoters. We tested for mitochondrial localization of the identified TFs in human cells and assessed whether the binding sites were negatively selected and hence, likely functional. Our findings support our approach and strongly suggest that mtDNA functions are regulated by both mitochondria-dedicated and bona fide nDNA TFs.

## Materials and Methods

### Analysis of ENCODE ChIP-seq Data

A flow chart describing the scheme of our workflow is provided (supplementary fig. S1, Supplementary Material online). The ENCODE ChIP-seq BAM files (hgdownload-test.cse.ucsc.edu/goldenPath/hg19/encodeDCC/, last accessed September 27, 2014) were downloaded and the mtDNA reads were sorted out and analyzed by Quantitative Enrichment of Sequence Tags—QuEST (mendel.stanford.edu/SidowLab/downloads/quest/, last accessed September 27, 2014). First, we analyzed mtDNA ChIP-seq peaks using QuEST default parameters (chip enrichment = 30). To reduce the false discovery rate while focusing on high ChIP-seq enrichment sites, we defined mtDNA-binding sites as only those that were identified during this stage of analysis. Next, we reanalyzed all of the files using QuEST low-stringency parameters (chip enrichment = 10). This secondary low stringency analysis enabled the detection of experiments that were completely deprived of mtDNA ChIP-seq peaks, allowing these to be excluded from further analysis. ChIP-seq data were also visualized using the DNAnexus ChIP-seq analyzer (www.DNAnexus.com, last accessed September 27, 2014). This allowed for comparing the analysis of the TF ChIP-seq files versus control ChIP-seq files. Jun-D, c-Jun, and CEBPb were analyzed according to the following parameters: ChIP candidate threshold, 30; experiment to background enrichment, 3; library fragment sizes: 120 (Jun-D and CEBPb), 132 (c-Jun); kernel bandwidths: 40 (Jun-D and CEBPb), and 44 (c-Jun). In this step, we analyzed the entire genome (i.e., nDNA and mtDNA) and took into account only those mtDNA peaks with *q* values found in the first percentile of all peaks. As mtDNA is a circular molecule, we analyzed the ChIP-seq peaks using two mtDNA references, namely the revised Cambridge Reference Sequence (GenBank number NC_012920) (Andrews et al. 1999) and the same sequence in which nucleotide positions 1–600 were removed and pasted at the end of the sequence.

### Analysis of ENCODE DNAse-seq BAM Files

The ENCODE digital genomic footprinting file of the HepG2 and IMR90 cell line (hgdownload-test.cse.ucsc.edu/goldenPath/hg19/encodeDCC/, last accessed September 27, 2014) was downloaded and the mtDNA-mapped reads were retrieved. Using MitoBAM-Annotator (Zhidkov et al. 2011), the number of reads in each position was counted. Hypersensitivity sites were identified using an algorithm that was recently proved successful for the identification of such sites in human mtDNA (Mercer et al. 2011) with the following specific parameters: Briefly, for each position in the mtDNA, an *F* score was calculated in sliding read windows of 20 bp, a value corresponding to the median of the previously used window size (Mercer et al. 2011). For the identification of DNase1-hypersensitive sites, regions of 60 bp in length were evenly divided into proximal, central, and distal fragments while highlighting sites having the lowest read counts in the central fragment. To this end, the following equation was applied: F = (C + 1)/L + (C + 1)/R, where C represents the average number of read in the central fragment, L represents the average read count in the proximal fragment, and R represents the average read count in the distal fragment. The lowest retrieved *F* scores across regions throughout the mtDNA were interpreted as hypersensitivity sites.

### Analysis of ENCODE RNA-seq Data of c-Jun, Jun-D, and CEBPb

Briefly, we downloaded and calculated uniformly processed, gene level expression estimates (in RPKM, i.e., reads per kilobase per million) from the ENCODE RNA portal (http://genome.crg.es/encode_RNA_dashboard/hg19/, last accessed September 27, 2014) for whole-cell PolyA+ RNA-seq data sets from the CSHL production group for five cell lines, namely HeLa-S3, K562, H1-hESC, HepG2, HUVEC, and IMR90. We extracted expression level data for c-Jun, Jun-D, and CEBPb from these files. For some cell lines that had expression estimates for two biological replicates, we averaged the RPKM values. We also obtained the total number of ChIP-seq-binding sites for the tested TFs in HeLa-S3, K562, H1-hESC, HepG2, HUVEC, and IMR90 cells using the ENCODE uniform ChIP-seq processing pipeline (Landt et al. 2012). Briefly, we obtained reproducible and rank-consistent peaks between replicate experiments by using the SPP peak-caller (Kharchenko et al. 2008) within the Irreproducible Discovery Rate framework (Qunhua et al. 2011). The ratio between mtDNA and nDNA reads was calculated by counting the reads within the ten most prominent binding peaks identified by the ENCODE consortium for each of the three tested TFs. Then, for each factor, we divided the number of mtDNA reads in the relevant peaks by the mean number of reads in nDNA sites.

### Bioinformatics Screen for TF mtDNA-Binding Motifs

To identify TF-binding motifs throughout the mtDNA, we subjected the mtDNA revised Cambridge Reference Sequence (NC_012920.1) to analysis by JASPAR (JASPAR.genereg.net/cgibin/, last accessed September 27, 2014), using the default parameters. We also used JASPAR to assess the effect of population genetics variants (see below) on the prediction score of motifs within ChIP-seq positive and ChIP-seq negative sites throughout the mtDNA (also see next subsection).

### Analysis of Tip and Nodal Mutational Events in Predicted and Experimentally Validated TFBS

**Fig. 1.—:**
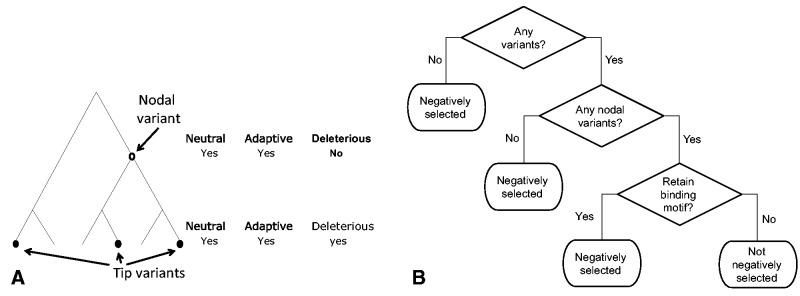
(*A*) A schematic phylogenetic tree representing nodal and tip variants. Tip variants, filled circles; nodal variants, open circles. (*B*) Decision tree describing the approach to identify negative selection in TFBS.

Nodal mutations in the phylogenetic tree are older and thus have a higher opportunity to have been affected by selection. Mutations appearing at the tips of the phylogenetic tree, appear in single individuals, are considered younger and hence have yet to undergo selection, as opposed to nodal events (Templeton 1996; Zhang et al. 2003; Ruiz-Pesini et al. 2004) (fig. 1*A*). To classify the variant events occurring within the identified TFBS into tips and nodal variants, we used our previous phylogenetic analysis of 9,868 whole mtDNA sequences representing all major global populations (Levin et al. 2013). As subsequent analysis was aimed to assess the effect of natural selection on the TFBS rather than on the protein-coding sequences that harbored them, human population variants were listed within the predicted binding sites, focusing only on those occurring at the third codon positions (using JASPAR, as mentioned above). For each predicted binding site, we listed the nodal and tip events according to previously published parameters (Levin et al. 2013). Briefly, we defined nodal events as variants which were shared by at least five individuals within the same phylogenetic lineage, and comprise at least 85% of the individuals of this sublineage. Tip events are variants which were identified only in single individuals and were not shared with other individuals in the same lineage. For each TF, we compared the tips and nodal events in our ChIP-seq-validated binding sites versus ChIP-seq negative sites harboring predicted TF-binding motifs. Three cases could be envisioned when using genetic variation to assess the signatures of negative selection at TFBS (fig. 1*B*): 1) The tested nucleotide positions at TFBS harbor no human genetic variants. In such a case, negative selection is likely the strongest. 2) The tested nucleotides harbor only tip variants. 3) The tested binding sites harbored nodal variants, which did not disrupt the prediction score of the TF-binding motif.

### Cells and Media

HepG2 cells were grown in 45% HAM-F12, 45% low glucose (1 g/l) Dulbecco’s modified Eagle’s medium (DMEM), 10% fetal calf serum (FCS), 2 mM l-glutamine, and 1 mM penicillin–streptomycin. Primary foreskin cells were grown in 90% high glucose (4.5 g/l) DMEM, 10% FCS, 2 mM l-glutamine, and 1 mM penicillin–streptomycin. H9-hESC cells were grown with 85% knockout DMEM F12, 15% FCS, nonessential amino acids, beta-mercaptoethanol, penicillin–streptomycin, and basic fibroblast growth factor. All cell lines were grown in a standard cell culture incubator with 5% CO_2_ at 37 °C.

### Subcellular Fractionation

Forty-eight hours after thawing, HepG2 cells, grown and harvested from 250-ml flasks, were washed twice with phosphate buffer saline (PBS) and resuspended in 1 ml fractionation buffer (250 mM sucrose, 20 mM HEPES, pH 7.4, 10 mM KCl, 1.5 mM MgCl_2_, 1 mM ethylenediaminetetraacetic acid, 1 mM ethylene glycol tetraacetic acid (EGTA), 1 mM DL-Dithiothreitol (DTT), and proteinase inhibitor cocktail [Sigma, #P2714]). The cell lysate was passed through a 25G needle five times and incubated on ice for 20 min, followed by a 7-min 720 × g centrifugation at 4 °C. The nuclear pellet was rewashed with 700 µl fractionation buffer and passed through a 25G needle as above. After a second 10-min centrifugation at 500 × g, the pellet was resuspended with 700 µl fractionation buffer, and again passed through a 25G needle. The solution was centrifuged (600 × g, 4 °C, 10 min) and the pellet was resuspended in lysis buffer (10% glycerol, 25 mM NaCl, 50 mM NaF, 10 mM napyrophosphate, 2 nM EGTA, 2 nM DTT, 20 nM p-nitrophenyl phosphate (pNPP), 25 mM Tris–HCl, pH 7.4, 50 nM beta-glycerolphosphate and 0.1% Triton X-100) to collect the nuclear fraction. The upper 700 µl from the first collected supernatant was centrifuged at 10,000 × g for 12 min at 4 °C and the remaining upper 500 µl was considered as the cytosolic fraction. The pellet, resuspended in lysis buffer, was considered as the mitochondrial fraction. This protocol was based on a previously published procedure (She et al. 2011).

### Western Blot and Antibodies

Equal amounts of protein (up to 21 µg) from the nuclear, cytosolic, and mitochondrial fractions were loaded onto a 10% sodium dodecyl sulfate polyacrylamide gel electrophoresis gel. The gel was blotted onto a polyvinylidene fluoride membrane (Immobilon, Millipore) for 1 h (mini-PROTEAN tetracell, BioRad), following the manufacturer’s protocol. After washing with PBST (phosphate-buffered saline Tween-20, for c-Jun or Jun-D) or TBST (Tris-buffered saline Tween-20 for VDAC1, Histone H3) and blocking with PBST or TBST containing 5% skim milk, the membrane was incubated for 1–1.5 h with antihistone-H3 (Santa Cruz-8654, 1:5,000) and anti-VDAC1 (Abcam, 1:1,000) antibodies targeted to nuclear and mitochondrial markers, respectively. Antibodies raised against c-Jun (Santa Cruz-1694, 1:1,000) and Jun-D (Santa Cruz-74, 1:3,000) were used to assess the subcellular localization of these proteins. After three PBST or TBST washes of 5 min each, the membrane was incubated for an hour with the horseradish peroxidase (HRP)-conjugated goat antirabbit (c-Jun, Jun-D), rabbit antigoat (Histone H3), or goat antimouse (VDAC1) secondary antibodies and washed three times with PBST or TBST. Finally, the membrane was incubated with an enhanced chemiluminescent HRP substrate solution (Millipore) for 5 min and subjected to image analysis (luminescent image analyzer LAS-3000, Fujifilm).

### Immunogold Labeling and Electron Microscopy

The postembedding immunoelectron microscopy protocol employed was a modified version of that described by Tokuyasu (1986). Briefly, HepG2 cells were fixed with 2% paraformaldehyde, 0.25% glutaraldehyde in 0.1 M phosphate buffer for 10 min at room temperature. The buffers were removed and the cells were incubated in fresh fixation solution for 2.5 h at 4 °C. The fixed cells were transferred to 2.3 M sucrose in PBS overnight. Cryosections were generated using a Leica UltraCut UCT ultramicrotome equipped with a low-temperature sectioning system. The sections were collected on Formvar/Carbon-coated nickel grids. The grids were blocked using PBG buffer (0.5% bovine serum albumin (BSA), 0.01 M glycine in PBS) for 10 min and incubated with the antibodies of interest (1:25–1:50 dilution) for 30 min. After five 2-min washes (1 × PBS, room temperature), the grids were incubates with 12-nm colloidal gold-conjugated goat antirabbit IgG (Jackson Laboratories; 1:20 or 1:40 dilution) for 30 min followed by five 2-min washes (1 × PBS). The grids underwent postfixation treatment with 1% glutaraldehyde in PBS for 10 min and were neutralized by 2% uranyl acetate (UA) and floated on 2% methyl cellulose (nine parts) supplemented by 3% UA (one part). Digital images were collected with a Gatan 830 ORIUS SC200 CCD camera and digital micrograph software. The cytoplasmic distribution of gold particles was assessed for each grid, and was first compared with the same distribution in the control experiment (secondary antibodies only). To calculate the probability of mitochondrial localization, we counted the mitochondrial gold labeling and compared them to the cytoplasmic labeling in multiple images of the experiment versus the control (chi-square test). Notably, we restricted our analysis only to images from cells that contained clear nuclear labeling of the TF antibodies and clear mitochondrial morphologies.

### Chromatin Immunoprecipitation

ChIP was performed as previously described (Sailaja et al. 2012; Gokhman et al. 2013) with 1–1.5 million cells and specific antibodies to c-Jun (Santa Cruz-1694), Jun-D (Santa Cruz-74), and CEBPb (Santa Cruz-150).

### Chromatin Immunoprecipitation-Quantitative Polymerase Chain Reaction

The identified mtDNA-binding sites for c-Jun and Jun-D were verified by ChIP-quantitative polymerase chain reaction (qPCR). qPCR primers were designed to flank the most prominent peaks and control regions (negative controls) encompassing mtDNA sites that did not harbor any detectable peaks (supplementary table S1, Supplementary Material online).

## Results

### ENCODE ChIP-seq Experiments Reveal Binding of Human mtDNA by c-Jun, Jun-D, and CEBPb

**Fig. 2.—:**
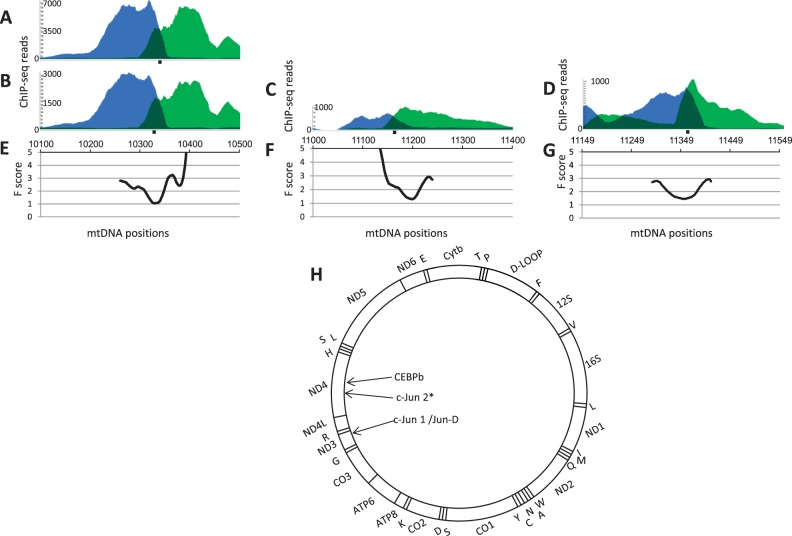
ChIP-seq peaks correspond to DNase1 hypersensitivity sites in human cells. *x* axis: mtDNA nucleotide positions. *y* axis: (*A–D*) The blue area represents positive strand reads, whereas the green area corresponds to reads from the negative strand. The dark green area designates the overlapping region between reads from the two strands. Black rectangle below the panel—JASPAR predicted TF-binding motif. *y* axis: (*E–G*) The *F* score (see Materials and Methods) of each mtDNA position in DNAse-seq experiments; the lower the score the more protected is the DNA by proteins. (*A*) Jun-D, (*B*) c-Jun site 1 (bind1), (*C*) c-Jun site 2 (bind2), (*D*) CEBPb, (*E*, *F*) DNase-seq of HepG2 cells, (*G*) DNase-seq of IMR90 cells. (*H*) A scheme summarizing the identified mtDNA-binding sites of c-Jun, Jun-D, and CEBPb. D-Loop—the main noncoding region. *12S, 16S*—rRNA genes. Capital letters—tRNAs genes. *ND* genes*, CO1-3, Cytb,* and *ATP6/8*—protein-coding subunits of OXPHOS complexes 1, 3, 4, and 5. Arrows point to the binding sites of the relevant TFs; asterisk, c-Jun-binding site 2.

As the first step, we used ENCODE consortium ChIP-seq data to map the physical binding of 168 TFs across the human genome in six human cell lines. Sequence reads mapped to human mtDNA were included in the so-called ENCODE “black list” as “these regions tend to have a very high ratio of multimapping to unique mapping reads and high variance in mapability” (http://genome.ucsc.edu/cgi-bin/hgFileUi?db=hg19&g=wgEncodeMapability, last accessed September 27, 2014). Therefore, to utilize ENCODE ChIP-seq data to detect candidate TFBS in human mtDNA, we used highly stringent peak-calling criteria so as to increase accuracy and specificity at the expense of sensitivity. To this end, we analyzed the entire ENCODE data set (780 ChIP-seq files) with the QuEST peak caller, using moderate identification criteria (chip enrichment = 30) (Valouev et al. 2008). This approach enabled the identification of the expected bimodal ChIP-seq pattern in two mtDNA sites for three TFs, c-Jun, Jun-D, and CEBPb (fig. 2*A*–*D* and table 1). Notably, the peak-caller used by ENCODE (SPP) also identified these binding sites, both located outside of the mtDNA D-Loop within the region encoding for the proteins *ND3* (Jun-D and c-Jun) and *ND4* (CEBPb) (fig. 2*H*). A bioinformatics screen for TF-binding motifs (JASPAR) predicted motifs that precisely overlapped our identified ChIP-seq signals, thus offering support for the TFBS identified here (table 1). While applying the low-stringency QuEST parameters for the ChIP-seq experiments of the three identified TFs (chip enrichment = 10; see Materials and Methods), an additional yet weak c-Jun-binding site was identified within the *ND4* gene (fig. 2 and table 1). We compared the ChIP-seq background signals around the mtDNA sites identified in this study with the ten most prominent ENCODE ChIP-seq nDNA signals reported for each of the tested three TFs (genome.ucsc.edu/ENCODE, last accessed September 27, 2014). We noticed that the level of mtDNA ChIP-seq background signal was 102-fold (Jun-D, SD = 10.42), 174-fold (c-Jun, SD = 16.68), and 15-fold (CEBPb, SD = 2.22) higher than that of the mean nDNA signals. Nevertheless, the mtDNA ChIP-seq peaks associated with the binding sites of all three factors were at least an order of magnitude higher than the background level and were consistent among replicated ENCODE experiments in the same cell line, supporting the validity of these sites. As ENCODE also performed genome-wide DNase1 sensitivity assays (DNAse-seq), we analyzed the available data for mtDNA and identified prominent hypersensitive sites overlapping the ChIP-seq peaks of all three TFs (fig. 2*E*–*G*). This suggests alteration of the mitochondrial nucleic acid–protein structure (i.e., the nucleoid) (reviewed in Kukat and Larsson 2013) around the identified mtDNA-binding sites.

**Table 1:** Summary of ENCODE ChIP-seq Screen

TF	mtDNA Position^a^	*Q* Value (−log)	Positive Cell Lines	Negative Cell Lines	JASPAR Motif mtDNA Positions
Jun-D	10337	235,239	HepG2, SkNsH^b^	K562, Hela, H1-hESC, Gm12878	10336–10346
c-Jun	10337	144,990	HepG2, Huvec^b^	K562, Hela, H1-hESC	10317–10329
	11177	12,433.1			11161–11173
CEBPb	11345	46,217.7	IMR90	H1Hesc, Ecc1, Gm12878, Hct116, K562, Mcf7	11351–11361

Note.—Positive cell lines, cell lines with positive ChIP-seq signals. Negative cell lines, cell lines lacking ChIP-seq signals.

^a^mtDNA nucleotide position in the center of the analyzed binding site.

^b^ChIP-seq peaks identified using low stringency search parameters in QuEST.

Notably, ChIP-seq signals of c-Jun, Jun-D, and CEBPb were only observed in some of the cell lines tested and not in others (even while using low stringent peak-calling parameters), suggesting tissue-specific mtDNA binding (table 1). The aforementioned high background level of the ChIP-seq and DNAse-seq data enabled us to reconstruct the entire mtDNA sequence of these cell lines. This revealed no cell line-derived sequence variability within the identified binding sites, thus excluding sequence variation as explaining the observed deviation in mtDNA binding among the tested cell lines.

To test for the possible contribution of TF expression level differences among cell lines to their pattern of DNA binding, we used ENCODE RNA-seq data to compare the levels of c-Jun, Jun-D, and CEBPb expression in six available cell lines (supplementary table S2, Supplementary Material online). For instance, analysis of H1-hESC and HepG2 cells revealed high levels of both c-Jun and Jun-D expressions (supplementary fig. S2 and table S2, Supplementary Material online), although c-Jun expression in HepG2 was notably higher, as compared with H1-hESC cells (∼4-fold). In contrast, Jun-D expression levels did not notably differ between these two cell lines (supplementary table S2, Supplementary Material online). Nevertheless, we did not discern any monotonic relationship between expression levels and binding capacity of any of the three tested TFs (as measured by the total number of genome-wide ChIP-seq peaks) across the different cell lines (supplementary table S2 and fig. S3, Supplementary Material online). Hence, cell line-derived differences in the expression levels of c-Jun, Jun-D, and CEBPb cannot alone explain the variability in mtDNA binding of these TFs in human cells.

### TF-Binding Occurs within the Cytoplasmic mtDNA and Not in nDNA Mitochondrial Pseudogenes

During evolution mtDNA fragments were transferred and integrated into the human nDNA thus creating Nuclear Mitochondrial pseudogenes (NUMTs mtDNA) (Tourmen et al. 2002; Woischnik and Moraes 2002; Hazkani-Covo et al. 2003; Mishmar et al. 2004). Sequence similarity of such NUMTs to the cytoplasmic mtDNA raises the possibility that some of the ChIP-seq reads originate from NUMTs, thus potentially causing erroneous mtDNA ChIP-seq signals. In order to control for such errors, we used only uniquely mapped ChIP-seq reads, thus excluding fragments exhibiting dual genomic/mtDNA localization. Additionally, we subjected the mtDNA sequences corresponding to the TFBS regions (±50 bp) to BLAST search the human genome database. The nDNA BLAST hits with the highest identity scores were aligned against the mtDNA of the cell lines in which ChIP-seq signals were identified. This analysis revealed a number of mutations, which clearly distinguished the most identical NUMT hits from their corresponding mtDNA TFBS (Jun-D—eight mutations, c-Jun—eight mutations for binding site 1, and three mutations for binding site 2, CEBPb—seven mutations). An analysis of the ChIP-seq reads revealed that the vast majority of all reads was identical to the cytoplasmic mtDNA sequence of the tested cell lines (tables 2–4). Specifically, the number of NUMT reads was extremely low, with a mean of 1.5 ± 1 SD out of a mean of 8,009 ± 1,354 SD read coverage for the positions encompassing the Jun-D-binding site, 1.6 ± 1.5 NUMT reads out of 3,841 ± 668 SD covering c-Jun-binding site 1 and no NUMT reads out of 893.8 ± 197 SD covering c-Jun-binding site 2 and a mean of 0.21 NUMT reads ± 0.36 SD out of 851 ± 362 SD total reads encompassing the CEBPb-binding site being noted (supplementary fig. S4A–C, Supplementary Material online). There was one nucleotide position which was identical between the nDNA hit and the mtDNA sequence of HepG2 (table 2 variant 8 and supplementary fig. S4, Supplementary Material online). Notably, this particular change (G > A at mtDNA position 10373) was outside of the predicted binding motif and is a known common mtDNA variant in the human population (Levin et al. 2013). We further corroborated this interpretation by the analysis of nearly the entire sequence of the mtDNA of HepG2 cells (∼78%), reconstructed from the HepG2 DNAse-seq reads (supplementary fig. S5, Supplementary Material online, coverage of >1,000× in 12,892 mtDNA positions) and defined its mtDNA haplotype. Furthermore, this variant appeared on reads that harbored another variant (mtDNA position 10370; coverage >4,700×; and see supplementary fig. S4, Supplementary Material online), which only appeared in the HepG2 mtDNA sequence and not in the nDNA hit. Therefore, this variant likely corresponds to the mtDNA haplotype of HepG2 cells Kloss-Brandstatter et al. (2011) rather than to the NUMT. We, therefore, conclude that the ChIP-seq reads encompassing the identified binding sites correspond to the active mtDNA rather than to NUMTs.

**Table 2:** ChIP-seq Reads Correlate with the Active mtDNA: mtDNA-Binding Site of Jun-D and Site 1 of c-Jun

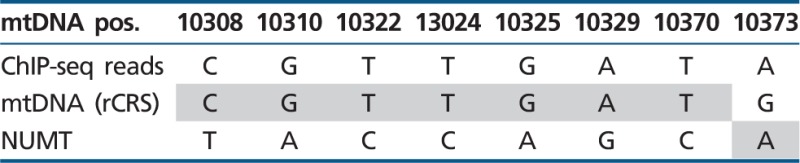

Note.—mtDNA pos, mtDNA nucleotide positions; ChIP-seq reads, the most common variant in the tested mitochondrial ChIP-seq-binding sites; mtDNA (rCRS), the identified variant within the revised Cambridge Reference Sequence (rCRS); NUMT, the variant found in the nDNA NUMT sequence with the highest BLAST score.

**Table 3:** ChIP-seq Reads Correlate with the Active mtDNA: mtDNA-Binding Site 2 of c-Jun

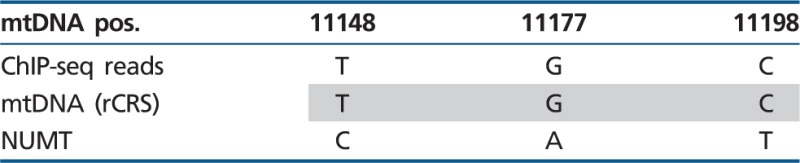

Note.—mtDNA pos, mtDNA nucleotide positions; ChIP-seq reads, the most common variant in the tested mitochondrial ChIP-seq-binding sites; mtDNA (rCRS), the identified variant within the revised Cambridge Reference Sequence (rCRS); NUMT, the variant found in the nDNA NUMT sequence with the highest BLAST score.

**Table 4:** ChIP-seq Reads Correlate with the Active mtDNA: mtDNA-Binding Site of CEBPb

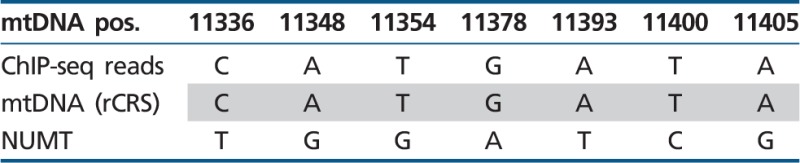

Note.—mtDNA pos, mtDNA nucleotide positions; ChIP-seq reads, the most common variant in the tested mitochondrial ChIP-seq-binding sites; mtDNA (rCRS), the identified variant within the revised Cambridge Reference Sequence (rCRS); NUMT, the variant found in the nDNA NUMT sequence with the highest BLAST score.

### mtDNA Binding by c-Jun and Jun-D Is Validated by ChIP-qPCR

**Fig. 3.—:**
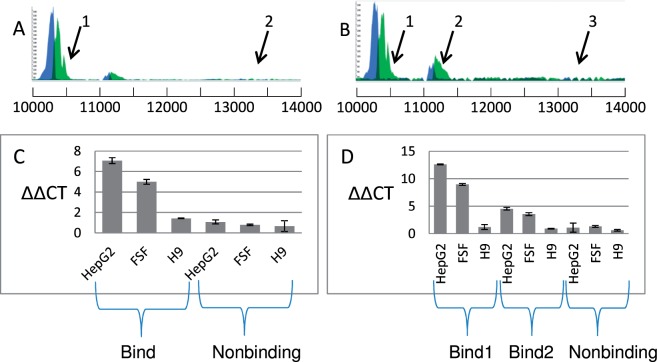
ChIP-seq validation experiment by ChIP-qPCR. ChIP experiments performed using HepG2, foreskin (FSF), and H9-hESC cells with anti-c-Jun or anti-Jun-D antibodies were followed by qPCR. (*A*) Jun-D-binding peaks. (1) The identified mtDNA-binding site. (2) mtDNA site lacking a ChIP-seq peak. (*B*) c-Jun-binding validation. (1) and (2) correspond to the identified mtDNA-binding sites, with (1) being the most prominent site. (3) mtDNA site lacking a significant ChIP-seq peak. (*C*) ChIP-qPCR signals for Jun-D. (*D*) ChIP-qPCR signals for c-Jun. Note that the PCR signal is stronger in the binding sites as compared with the negative controls (nonbinding sites and H9-hESC cells [H9]) that showed no binding.

To assess the validity of the identified ChIP-seq signals, we performed ChIP of both c-Jun and Jun-D (separately), followed by qPCR of each of the identified binding sites and control background mtDNA regions (fig. 3 and supplementary table S1, Supplementary Material online). These experiments were performed in cells exhibiting ChIP-seq peaks (e.g., HepG2), as well as in cells lacking such peaks (H9-hESC, a sister cell line of the H1Hesc cells used by ENCODE). Binding was also examined in early passage nonimmortalized human foreskin fibroblasts (FSF) to control for possible effects of cellular transformation. Considerable enrichment of the PCR signal was observed in the identified peak region of HepG2 and FSF cells but not in H9-hESC cells for both c-Jun and Jun-D (fig. 3). Interestingly, the signal identified in the most prominent mtDNA site (c-Jun bind1) was more than 2-fold higher than that of the second identified binding site (c-Jun bind2) both in the ENCODE ChIP-seq data and in the ChIP-qPCR experiments, reflecting the stronger binding capacity of c-Jun to the former site. Notably, in our hands ChIP-qPCR experiments did not lend support for the binding of CEBPb. Although we cannot rule out technical (antibody-related) issues, further experimental analysis was focused on c-Jun and Jun-D.

### c-Jun and Jun-D Are Localized to the Mitochondria in HepG2 Cells

**Fig. 4.—:**
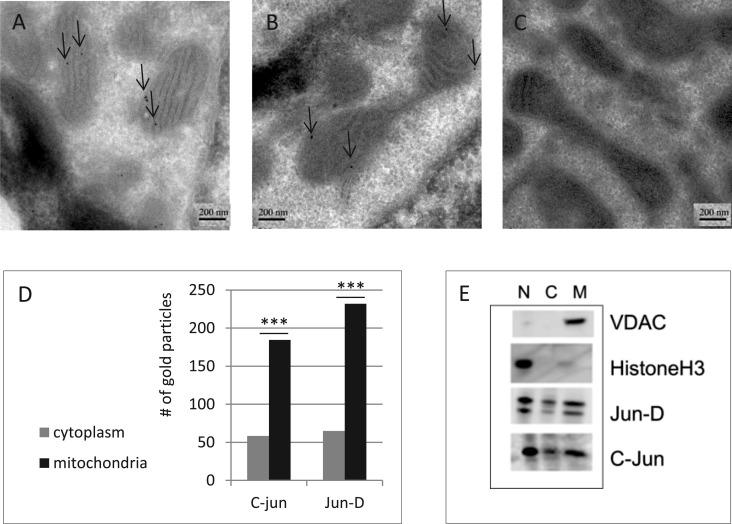
Mitochondrial localization of c-Jun and Jun-D in human cells. (*A*) Immunogold labeling of Jun-D and c-Jun in mitochondria of HepG2 cells. Mitochondria-associated signals are indicated by arrows. (*A*) Jun-D immunogold labeling. (*B*) c-Jun immunogold labeling. (*C*) A control experiment was conducted using secondary antibodies alone. (*D*) Quantification of mitochondrial particles (black) versus cytoplasmic particles (gray) reflecting antibody labeling of c-Jun and Jun-D. ***P value < 0.001. *y* axis: Number of gold particles, *x* axis: Analyzed proteins. (*E*) To assess the mitochondrial localization of c-Jun and Jun-D by subcellular fractionation, histoneH3 and VDAC1 were used as markers for the purity of the nuclear (N) and mitochondrial (M) fractions, respectively. The presented results summarize three independent fractionation experiments.

As the TFs identified in this study bind mtDNA in human cells at specific sites, we assessed their mitochondrial localization. Indeed, previous studies supported mitochondrial localization of one of these TFs, c-Jun, in rat (Haase et al. 1997). Accordingly, we considered the subcellular localization of both c-Jun and Jun-D by immunogold labeling as viewed by electron microscopy and by Western blot of protein extracts of various subcellular fractions. First, we sought these TFs in HepG2 cells in an immunogold labeling protocol, as revealed by electron microscopy (fig. 4). Notably, analysis was restricted only to cells with clear TF signal in the nucleus. To this end, we compared the number of gold particles, reflecting mitochondrial labeling by the anti-TF antibodies, with the number of gold particles reflecting cytoplasmic labeling by these antibodies, in multiple images and experiments (for details, see Materials and Methods). By comparing the extent of mitochondrial anti-TF antibody labeling with that obtained in a control experiment when only secondary antibodies were employed, we demonstrated statistically significant mitochondrial localization of both c-Jun and Jun-D (fig. 4*D*; analysis of at least 50 electron microscopy images per TF, chi square test, *P* < 0.001). To further assess the validity of this localization experiment, we performed Western blot analysis of equal amounts of protein extracts prepared from nuclear, cytosolic, and mitochondrial fractions using antibodies directed against nuclear (i.e., histone H3) and mitochondrial (i.e., VDAC1) markers. This experiment detected these proteins only in the nuclear and mitochondrial fractions, respectively. In contrast, antibodies against both c-Jun and Jun-D recognized these proteins in the mitochondrial, cytosolic, and nuclear fractions (fig. 4). As equal amounts of proteins were loaded onto the gel, the significantly stronger signal of the TF-mitochondrial signal, as compared with the cytoplasmic signal (three independent experiments) (supplementary fig. S6, Supplementary Material online, and fig. 4), is consistent with the electron microscopy experiments. Similar results were shown in primary human foreskin cells (not shown). We conclude that in HepG2 and primary foreskin cells, c-Jun and Jun-D are located preferentially in the nucleus and at the mitochondria.

### The Identified mtDNA TFBS Are under Selective Constraints

Signatures of selection are frequently used as a reflection of functionality in a given DNA sequence, including TFBS (Villar et al. 2014). Accordingly, recent analysis of human genetic variation patterns within genome-wide TFBS revealed signatures of negative selection (Khurana et al. 2013). These investigators compared identified ChIP-seq-binding sites with falsely predicted motifs of the studied TFs. We applied a similar logic to our identified c-Jun, Jun-D, and CEBPb mtDNA-binding sites, and assessed signatures of selection utilizing human genetic variation. In contrast to most genome-wide ChIP-seq sites, all of our identified binding sites were within mtDNA-encoded protein-coding genes. This raised the problem of differentiating the selective constrains acting on the amino acid sequences of the genes from those acting on the TFBS. In order to enrich for nucleotides that are under selective constrains acting on the TFBS, and to differentiate these constrains from those acting on the amino acid sequences of the genes harboring them (*ND4* and *ND3*), we focused our analysis solely on variants occurring at the third codon positions in each of the identified sites. We are aware of the possibility that such analysis cannot exclude selection for specific codons. Furthermore, for the sake of simplicity, we decided to focus our assessment on patterns of negative selection. Three types of genetic variation could show signatures of negative selection at TFBS: 1) The tested nucleotide positions at the TFBS harbor no human genetic variants; 2) the tested nucleotides harbor only tip variants; and 3) the tested binding sites harbored nodal variants, which retain the predicted TF-binding motif (see Materials and Methods and fig. 1*A* and *B*).

With this logic in mind, we used 9,868 human complete mtDNA sequences encompassing all major global populations that we recently analyzed (Levin et al. 2013) and recorded all genetic variants occupying the ChIP-seq positive binding motifs (CPRs), as well as predicted binding motifs that were in ChIP-seq negative regions (CNRs): *n* = 55 for Jun-D; *n* = 31 for c-Jun, and *n* = 74 for CEBPb. The total number of analyzed variants was 604 for Jun-D; 333 for c-Jun; and 852 for CEBPb (supplementary table S3, Supplementary Material online). Second, we divided the variants into those occurring at the tips (*n* = 396 for Jun-D; *n* = 282 for c-Jun; *n* = 564 for CEBPb) and nodes (*n* = 89 for Jun-D; *n* = 51 for c-Jun; *n* = 116 for CEBPb) of the human mtDNA phylogeny. Third, we calculated the effects of the variants on TF motif prediction scores (JASPAR). Finally, mtDNA variation patterns in predicted binding motifs, which are located within CNRs in the coding region, were compared with the CPRs.

**Fig. 5.—:**
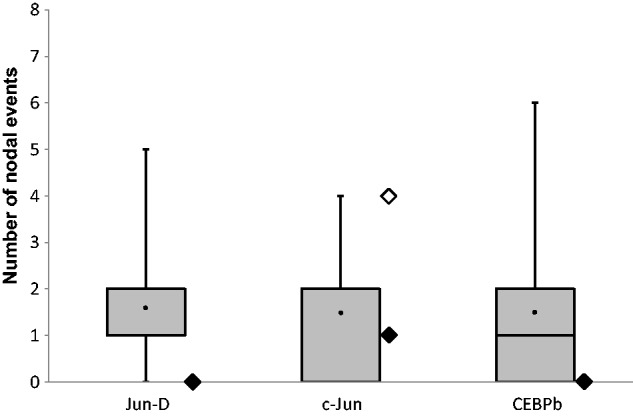
Number of nodal events in the binding sites of Jun-D, c-Jun, and CEBPb. Number of nodal events in the binding site of the identified TFs represented by filled rhombus; the weaker binding site of c-Jun—bind 2—is represented by an open rhombus. Distribution of nodal events within each of the predicted binding sites (CNRs) represented by box plots (note—the whiskers do not represent average and SD). *y* axis: Number of mutational events (variants); *x* axis: The analyzed TFs. We noticed that for Jun-D the median equals to the first quarterly and for c-Jun the median equal to the third quarterly. Filled circle, the average of nodal events in the CNRs of each TF.

**Fig. 6.—:**
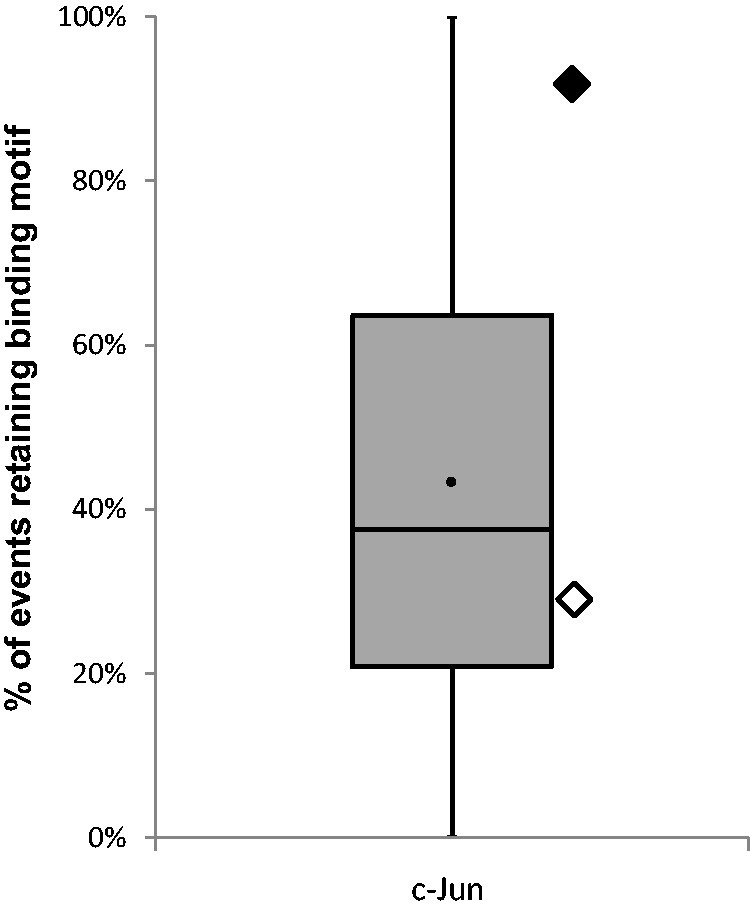
Percentage of variant events which retained the predicted binding motif of c-Jun. The strong binding site of c-Jun (bind 1) is represented by a filled rhombus and the weaker binding site of c-Jun (bind-2) is represented by an open rhombus. Nodal events do not change the prediction score in c-Jun bind 1, but change it in c-Jun bind 2.The distribution of the mutational events which retain the binding motif of the CNRs is represented by a box plot. *y* axis: percentage of mutational events which retain the binding motif prediction motif; *x* axis: TF.

In the case of Jun-D- and CEBPb-binding sites, in contrast to most CNRs, the CPRs harbored only tip variants (fig. 5 and supplementary table S3, Supplementary Material online). In the case of c-Jun, both CPRs harbored both nodal and tip variants (fig. 5 and supplementary table S3, Supplementary Material online). However, whereas the single nodal variant and most tip variants of “c-Jun bind 1” did not alter the binding motif prediction scores, the nodal and tip variants of “c-Jun bind 2” did reduce the scores below the prediction threshold in a similar manner to the CNRs, suggesting negative selection acting only on “c-Jun bind 1” (fig. 6 and supplementary table S4, Supplementary Material online). Notably, as shown above, “c-Jun bind 1” bound the mtDNA stronger as compared with “c-Jun bind 2” (fig. 3), thus suggesting that the stronger binding site is likely more functionally important than is the weaker site.

It is possible that some of the CNRs lie within true binding sites of other proteins that were not identified in this study. A recent analysis mapped DNAse1 sensitivity sites that harbor binding motifs of various TFs throughout the human mtDNA (Mercer et al. 2011). We found that indeed several of the CNRs (five in Jun D, one in c-Jun, and five in CEBPb) colocalized with such DNAse1 sites (supplementary table S3, Supplementary Material online), of which some (2/5 in JunD, 1 in c-Jun, and 2/5 in CEBPb) either had no variants or had only tip variants, which is consistent with the effect of negative selection, thus implying potential functionality.

Taken together, these findings reflect signatures of negative selection within the strong mtDNA CPRs, thus supporting their functionality.

## Discussion

Our work has revealed two major novel observations: 1) The discovery that the mtDNA-binding sites of three bona fide nDNA TFs (c-Jun, Jun-D, and CEBPb) occur within mtDNA genes and are negatively selected, thus providing first support for their functional importance; and 2) the identification and experimental validation of human mtDNA binding and mitochondrial localization of c-Jun and Jun-D in cells. Notably, the identified TFs (c-Jun, Jun-D, and CEBPb) are well-known modulators of nDNA-encoded genes, among which are those encoding proteins that are imported into the mitochondria where they are important for mitochondrial function (Brady et al. 1992; Xia et al. 1998; Yubero et al. 1998; Tsuji 2005). As transcriptional coexpression of genes coding for components of the OXPHOS system was identified in different tissues (van Waveren and Moraes 2008; Garbian et al. 2010), our findings lend further support to the thought that mitonuclear coregulation is plausible (Bar-Yaacov et al. 2012).

Our screen of the ChIP-seq data sets generated by the ENCODE consortium revealed the binding sites of three TFs, of which only two were experimentally validated. However, to avoid the false discovery rate, we used stringent criteria of existing peak-callers that were originally designed to identify TFBS in the nDNA. During the course of our work, a similar screen was performed using low stringent criteria than used by us, resulting in the identification of eight candidate mtDNA-binding TFs (Marinov et al. 2014). Similar to us, Marinov et al. not only identified the human mtDNA binding of c-Jun, Jun-D, and CEBPb but also identified the binding of five additional TFs. Moreover, they identified more CEBPb and c-Jun ChIP-seq peaks in the mtDNA than we identified. Neither of the additional c-Jun and CEBPb peaks nor the additional TFBS that Marinov et al. identified complied with our stringent screen criteria. As none of these binding sites was experimentally validated, some of them might be false positive. Our identification and experimental validation of the c-Jun- and Jun-D-binding sites, the discovery of their mitochondrial localization in human cells, and the finding that these binding sites are negatively selected constitute the first solid evidence for the functional importance of these sites. Nevertheless, aside from these compelling pieces of evidence, it is likely that our stringent criteria may overlook true binding sites, hence creating false negative results. Both our work and the study by Marinov et al. emphasize the need to develop a ChIP-seq peak-caller with identification criteria adapted to the mtDNA. Future improvements in our definition of peak-calling criteria for mtDNA analysis may increase the sensitivity of our search while maintaining specificity. Such modifications may serve to expose a more complete range of candidate TFs involved in mtDNA transcription regulation.

In this work, we provide evidence for negative selection acting on the identified ChIP-seq-binding sites of c-Jun (bind 1), Jun-D, and CEBPb. This, for the first time, supports the functional importance of these sites, although the nature of this functionality has yet to be deciphered (Graur et al. 2013). In brief, the role of these TF binding could range from involvement in transcription regulation, signals for higher order mtDNA structure or even flags for the binding of other TFs as was recently shown for AP1 (c-Jun), which generated potentiation of chromatin accessibility for glucocorticoid receptor binding (Biddie et al. 2011). We noticed that the binding sites of c-Jun, Jun-D, and CEBPb clustered around the *ND4–ND3* genes, implying the possible existence of regulatory elements in this particular region. Although nontranscription functions could be caused by TF binding (mentioned above), this observation further supports the hypothesis that mtDNA transcriptional regulation is more complex than once assumed and that it is not solely regulated by mitochondria-dedicated factors. This also suggests that similar to a recent discovery in the nDNA, human mtDNA-coding sequences serve dual roles, namely coding for genes but also possessing regulatory potential (Birnbaum et al. 2012; Stergachis et al. 2013).

As site-directed mutagenesis is not currently possible for human mtDNA, we still cannot directly test the functional importance of mutations in our identified TFBS in cells. Moreover, no disease-causing mutations were reported in the nucleotide positions encompassing the validated binding sites of c-Jun and Jun-D, although our analysis of more than 9,868 whole mtDNA sequences representing a global human population revealed population genetic variants within the TFBS. As we have already shown that ancient mtDNA variants may affect mtDNA transcription regulation (Suissa et al. 2009), and as we found that some variants (tree tip variants) altered the binding motifs in our identified TFs, it is tempting to suggest that certain mtDNA variants in the discovered TFBS have functional potential.

Over the past few years, several bona fide nDNA TFs were identified in mitochondria of certain tissues (recently reviewed in Szczepanek et al. 2012; Bestwick and Shadel 2013). In contrast to our hypothesis-free TFBS screen, the discovery of mtDNA binding and determination of the subcellular localization of these additional TFs were motivated by prior interest in the function of these factors. Notably, one of these TFs (MEF2D) was not included in the ENCODE screen thus far. Another such TF, CREB, previously identified as mtDNA-bound in nonhuman mammals (De Rasmo et al. 2009), was not identified as mtDNA-bound in our screen of the ENCODE data set. Additionally, although the TFs RelA and STAT3, previously identified as being imported into human mitochondria (reviewed in Szczepanek et al. 2012), were included in the ENCODE data set, we did not identify any relevant significant ChIP-seq peaks in any of the tested cell lines. This could be partially due to the fact that mtDNA binding by RelA and STAT3 was observed in cell lines other that those tested by ENCODE.

We noticed that the observed mtDNA TFBS were identified in the exact same positions in the different cell lines tested. However, ChIP-seq peaks were not identified in all tested cell lines, thus implying the existence of cell type-specific patterns of mtDNA regulation. This is consistent with a recent mapping effort of DNaseI-hypersensitive sites within human mtDNA which also revealed cell line specificity in the occurrence of such sites (Mercer et al. 2011). Tissue-specific binding patterns of TFs could explain why c-Jun and Jun-D were not identified as mtDNA binders in many studies and instead, were frequently used as markers of nuclear localization. On the other hand, the glucocorticoid receptor was reported to bind mtDNA in HepG2 cells (Psarra and Sekeris 2011), although our analysis of the updated ENCODE data files did not identify such binding in other cell lines (e.g., the A549 and ECC1 lines). With this in mind, it is notable that mechanisms underlying tissue specificity of mitochondrial disorders have yet to be elucidated, although retrograde signaling was suggested to play a role (Raimundo et al. 2012). It would, therefore, be of interest to investigate the involvement of tissue-specific mtDNA transcription regulation in mitochondrial diseases.

## Conclusions

In this study, we employed a hypothesis-free approach to screen the entire ENCODE ChiP-seq data set, leading to the identification of in vivo binding sites of three TFs (c-Jun, Jun-D, and CEBPb) in human mtDNA. We identified two bona fide nuclear TFs (c-Jun and Jun-D) which localized to the human mitochondria and bound the mtDNA outside of the D-loop and relatively far from the major transcription regulatory elements. The identification of novel mtDNA binding by TFs that are long known regulators of nDNA genes raises the possible cotranscriptional regulation of the human nuclear and mitochondrial genomes and that mitochondrial transcription is not separately regulated but is rather part of the overall cellular regulatory machinery. Finally, the identified signatures of negative selection within the validated mtDNA TFBS support, for the first time, their functionality.

## Supplementary Material

Supplementary figures S1–S6 and tables S1–S4 are available at *Genome Biology and Evolution* online (http://www.gbe.oxfordjournals.org/).

Supplementary Data
