# Supplementary material for: Transcription Factors Bind Negatively Selected Sites within Human mtDNA Genes
Source: Genome Biol Evol. 2014 Sep 22;6(10):2634–46. doi: 10.1093/gbe/evu210 (PMC4224337; doi:10.1093/gbe/evu210)
Supplement: Supplementary Data [file supp_6_10_2634__index.html]

Transcription factors bind negatively-selected sites within human mtDNA genes — Transcription Factors Bind Negatively Selected Sites within Human mtDNA Genes — Supplementary Data 

# Transcription Factors Bind Negatively Selected Sites within Human mtDNA Genes

## Supplementary Data

files

**Files in this Data Supplement:**

- Supplementary Data - pdf file
- Supplementary Data - pdf file
- Supplementary Data - pdf file
- Supplementary Data - pdf file
- Supplementary Data - pdf file
- Supplementary Data - pdf file
- Supplementary Data - pdf file
- Supplementary Data - png file
- Supplementary Data - xlsx file
- Supplementary Data - xlsx file
