# Supplementary figures and images for: Transcription Factors Bind Negatively Selected Sites within Human mtDNA Genes
Source: Genome Biol Evol. 2014 Sep 22;6(10):2634–46. doi: 10.1093/gbe/evu210 (PMC4224337; doi:10.1093/gbe/evu210)

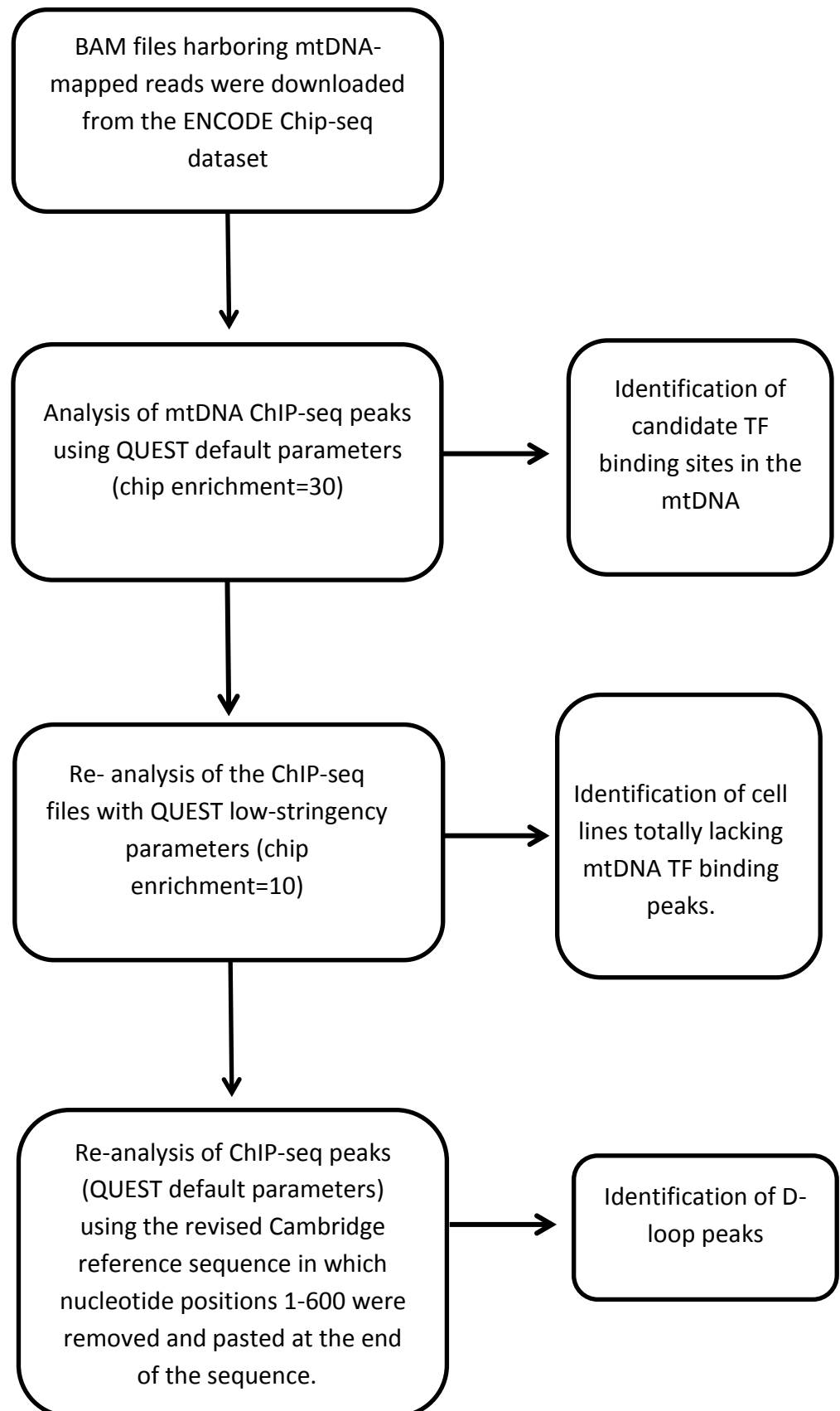

Supplement: Supplementary Data [file supp_evu210_supplemantary_figure_S1.pdf]

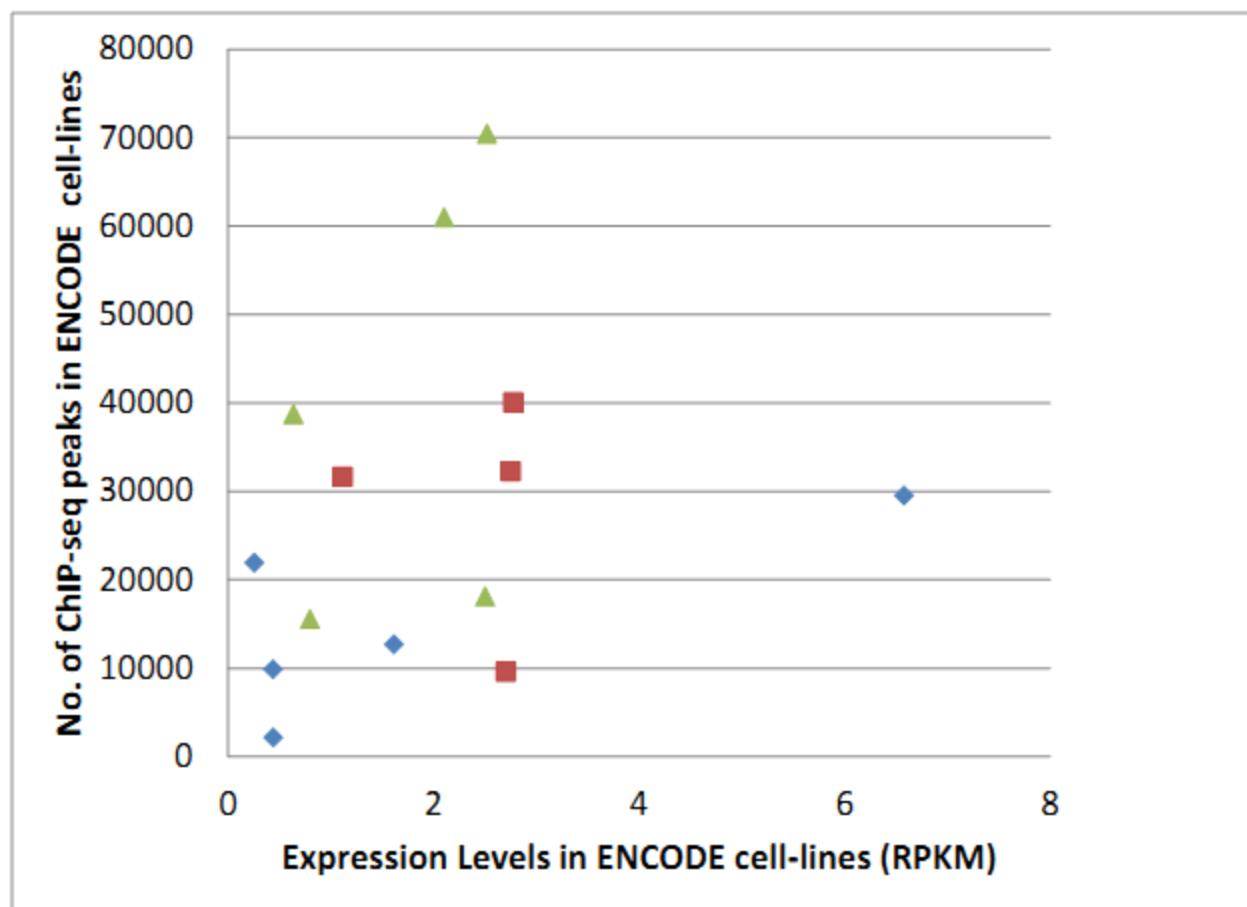

Supplement: Supplementary Data [file supp_evu210_supplemantary_figure_S3.pdf]

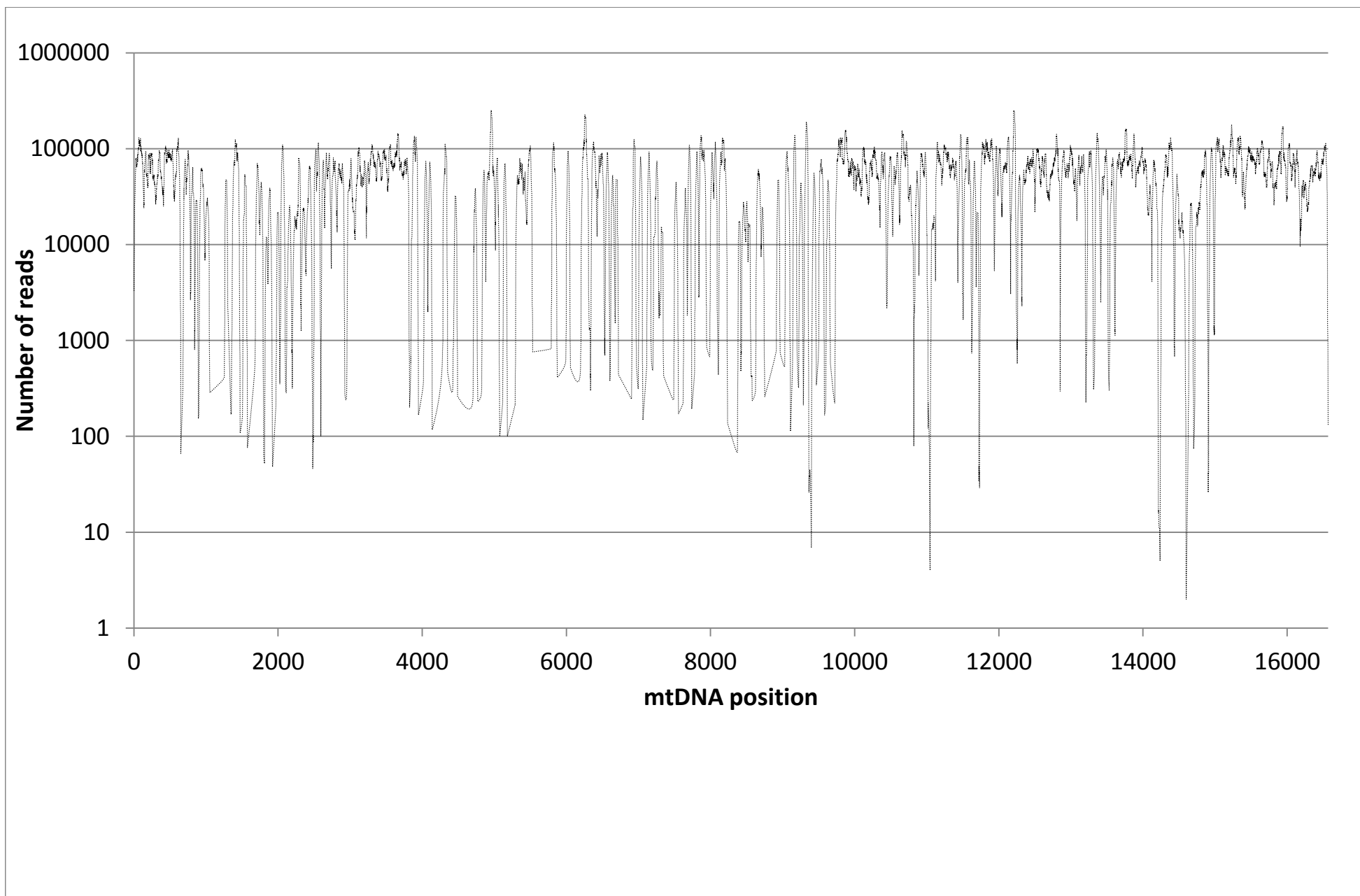

Supplement: Supplementary Data [file supp_evu210_supplemantary_figure_S5.pdf]

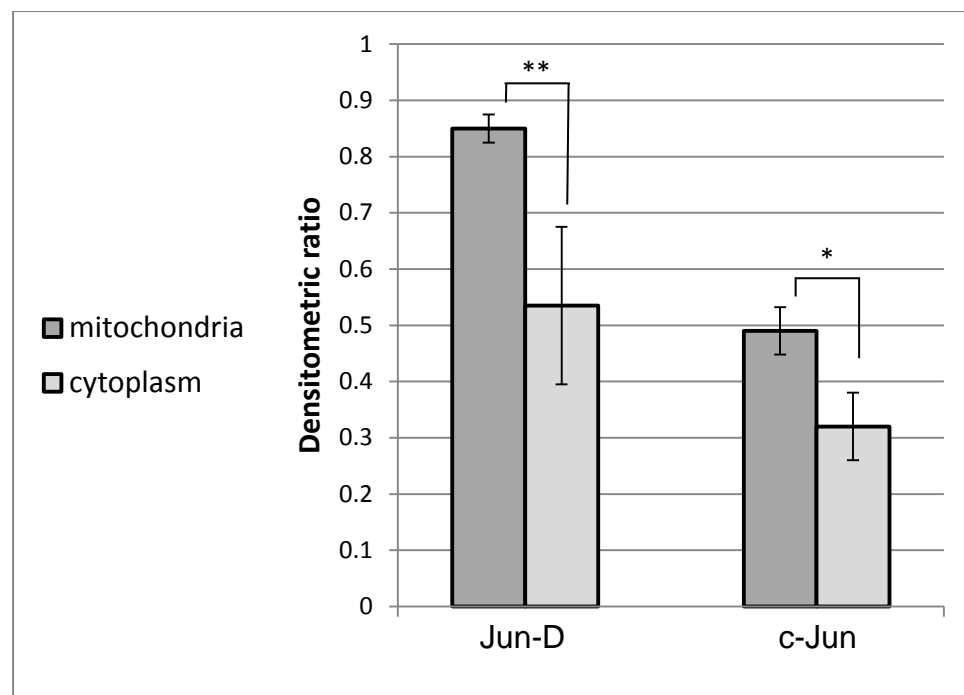

Supplement: Supplementary Data [file supp_evu210_supplemantary_figure_S6.pdf]

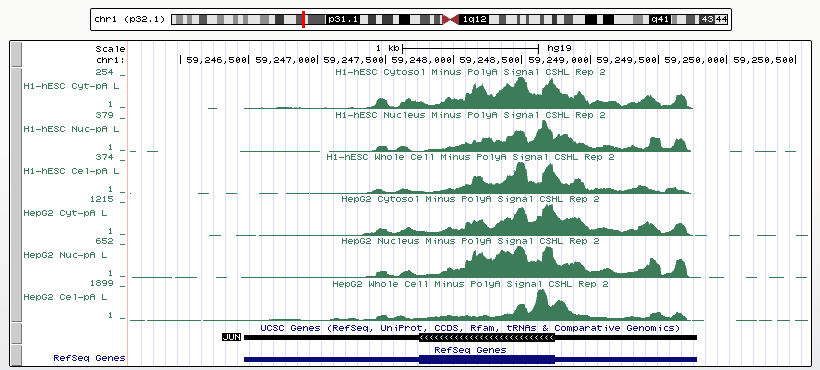

Supplement: Supplementary Data [file supp_evu210_supplemantary_figure_S2.png]
