# Supplementary material for: Transcription Factors Bind Negatively Selected Sites within Human mtDNA Genes
Source: Genome Biol Evol. 2014 Sep 22;6(10):2634–46. doi: 10.1093/gbe/evu210 (PMC4224337; doi:10.1093/gbe/evu210)
Supplement: Supplementary Data [file supp_evu210_supplemantary_figure_S4.pdf]

A.

Jun-D and c-Jun site 1

```
                           1 2           3 45  6                                        7  8
mtDNA 10284    CCATGAGCCCTACAAACAACTAACCTGCCACTAATAGTTATGTCATCCCTCTTATTAATCATCATCCTAGCCCTAAGTCTGGCCTATGAGTGA  10376
               |||||||||||||||||||||||| | ||||||||||| |  || ||||||||||||||||||||||||||||||||||||||||| || |||
NUMT (chr5)    CCATGAGCCCTACAAACAACTAACTTACCACTAATAGTCACATCGTCCCTCTTATTAATCATCATCCTAGCCCTAAGTCTGGCCTACGAATGA
```

Bind site of Jun-D in HepG2 rep1;rep2

	1	2	3	4	5	6	7	8
**MtDNA pos.**	10308	10310	10322	13024	10325	10329	10370	10373
**mtDNA**	C	G	T	T	G	A	T	G
**NUMT**	T	A	C	C	A	G	C	A
**A**	0;5	1;2	1;1	3;4	2;4	7889;9934 **G**	1;1	4794;5978 **A**
**C**	6456;7774 **C**	3;4	3;2	2;2	0;0	0;0	0;1	0;0
**G**	0;1	6867;8417 **G**	1;3	1;0	8089;9894 **G**	0;2	3;1	2;2
**T**	0;0	0;4	8334;10019 **T**	8118;9895 **T**	2;0	1;0	4706;5743 **T**	0;1

Site 1 of c-Jun in HepG2 rep1;rep2

	1	2	3	4	5	6	7	8
**MtDNA pos.**	10308	10310	10322	13024	10325	10329	10370	10373
**mtDNA**	C	G	T	T	G	A	T	G
**NUMT**	T	A	C	C	A	G	C	A
**A**	0;3	1;0	0;1	0;1	1;0	3067;5594 **A**	1;2	1950;3210 **A**
**C**	2396;4337 **C**	1;3	6;2	2;6	0;3	3;3	1;1	1;0
**G**	0;1	2593;4693 **G**	3;2	0;7	3078;5595 G	0;1	0;3	2;0
**T**	2;0	O;1	3116;5666 **T**	3072;5589 T	2;2	0;1	1896;3082 **T**	0;0

B.

c-Jun site 2

```
                                   1                                                 2                        3
mtDNA 11128   AACCACACTTATCCCCACCTTGGCTATCATCACCCGATGAGGCAACCAGCCAGAACGCCTGAACGCAGGCACATACTTCCTATTCTACACCCTAGTAGGC 11227
              |||||||||||||||||||| |||||||||||||||||||||||||||| |||||||||||||||||||| |||||||||||||||||||||||||||||
NUMT(chr5)    AACCACACTTATCCCCACCCTGGCTATCATCACCCGATGAGGCAACCAACCAGAACGCCTGAACGCAGGTACATACTTCCTATTCTACACCCTAGTAGGC
```

Site 2 of c-Jun in HepG2 rep1;rep2

	1	2	3
**MtDNA pos.**	11148	11177	11198
**mtDNA**	T	G	C
**NUMT**	C	A	T
**A**	0;0	0;0	0;0
**C**	0;0	0;0	861;1072 **C**
**G**	0;0	1026;1156 **G**	1;0
**T**	607;641 **T**	0;1	0;0

C.

CEBPb

```
                                                                1           2     3                       4              5      6    7
MtDNA 11304  ACTGCCCAAGAACTATCAAACTCCTGAGCCAACAACTTAATATGACTAGCTTACACAATAGCTTTTATAGTAAAGATACCTCTTTACGGACTCCACTTATGACTCCCTA 11411
             |||||||||||||||||||||||||||||||| ||||||||||| ||||| ||||||||||||||||||||||| |||||||||||||| |||||| |||| ||||||
NUMT (chr5)  ACTGCCCAAGAACTATCAAACTCCTGAGCCAATAACTTAATATGGCTAGCGTACACAATAGCTTTTATAGTAAAAATACCTCTTTACGGTCTCCACCTATGGCTCCCTA
```

Bind1 of CEBPb in Imr90 rep1;rep2

	1	2	3	4	5	6	7
**MtDNA pos.**	11336	11348	11354	11378	11393	11400	11405
**mtDNA**	C	A	T	G	A	T	A
**NUMT**	T	G	G	A	T	C	G
**A**	0;0	1215;1379 A	1;1	0;0	469;556 A	1;0	496;524 A
**C**	821;845 c	0;0	1;1	1;0	0;0	0;0	1;0
**G**	0;0	0;0	0;0	809;906 G	0;0	0;0	1;0
**T**	0;0	3;2	1382;1526 T	1;0	2;0	484;515 T	1;0
