# Supplementary material for: Transcription Factors Bind Negatively Selected Sites within Human mtDNA Genes
Source: Genome Biol Evol. 2014 Sep 22;6(10):2634–46. doi: 10.1093/gbe/evu210 (PMC4224337; doi:10.1093/gbe/evu210)
Supplement: Supplementary Data [file supp_evu210_supplemantary_table_S1.pdf]

Table S1. Primers for ChIP/qPCR

Primer name	Sequence 5`-3`	Position 5`-3` at the revised Cambridge Reference sequence (NC_012920.1)
c/D-Jun Bind-1 - Forward<br>c/D-Jun Bind-1 – Reverse	CCC TAC CAT GAG CCC TAC AA<br>TGT AAA TGA GGG GCA TTT GG	10279-10298<br>10485-10466
c-Jun Bind-2 – Forward<br>c-Jun Bind-2 – Reverse	CCA CAC TTA TCC CCA CCT TG<br>GAG TAG GGG AAG GGA GCC TA	11130-11149<br>11242-11223
c/D-Jun Nonbinding-1- Forward<br>c/D-Jun Nonbinding-1 – Reverse	ATG CTT AGG CGC TAT CAC CAC TCT<br>GTG TGG TTG GTT GAT GCC GAT TGT	13170-13193<br>13305-13282
c/D-Jun Nonbinding-2 – Forward<br>c/D-Jun Nonbinding-2 – Reverse	ATT GGC AGC CTA GCA TTA GCA GGA<br>TTC GAG TGC TAT AGG CGC TTG TCA	13459-13482<br>13612-13589
