# Supplementary material for: Transcription Factors Bind Negatively Selected Sites within Human mtDNA Genes
Source: Genome Biol Evol. 2014 Sep 22;6(10):2634–46. doi: 10.1093/gbe/evu210 (PMC4224337; doi:10.1093/gbe/evu210)
Supplement: Supplementary Data [file supp_evu210_supplemantary_table_S2.pdf]

Cell line	c-Jun expression (RPKM)	No. of c-Jun ChIP-seq peaks	Jun-D expression (RPKM)	No. of Jun-D ChIP-seq peaks	CEBPB expression (RPKM)	No. of CEBPb ChIP-seq peaks
HeLa-S3	0.26	21903	1.11	31633	2.10	61004
K562	0.44	9848	2.77	40052	0.64	38715
H1-hESC	0.44	2148	2.70	9550	0.78	15557
HepG2	1.61	12672	2.75	32275	2.499	18125
HUVEC	6.58	29524	4.45	N/A	0.32	N/A
IMR90	N/A	N/A	N/A	N/A	2.52	70445

Table S2: Expression levels (RPKM) and number of reproducible ChIP-seq peaks of c-Jun, Jun-D and CEBPb in five cell lines studied by ENCODE.
